# Supplementary material for: The Occurrence of Antimicrobial-Resistant Salmonella enterica in Hatcheries and Dissemination in an Integrated Broiler Chicken Operation in Korea
Source: Animals (Basel). 2021 Jan 11;11(1):154. doi: 10.3390/ani11010154 (PMC7827806; doi:10.3390/ani11010154)
Supplement: Supplementary file 1 [file animals-11-00154-s001.zip › supple/Figure S2,Table S1-S4.docx]

**Figure S2**. The prevalence of *Salmonella* isolates from downstream of the hatchery step along the integrated broiler chicken operation, with overlap of the PFGE type with hatchery (see the details in Table 6). Backgrounds with the same colored bars (orange) indicate samples with the same PFGE patterns and antimicrobial resistance profiles.
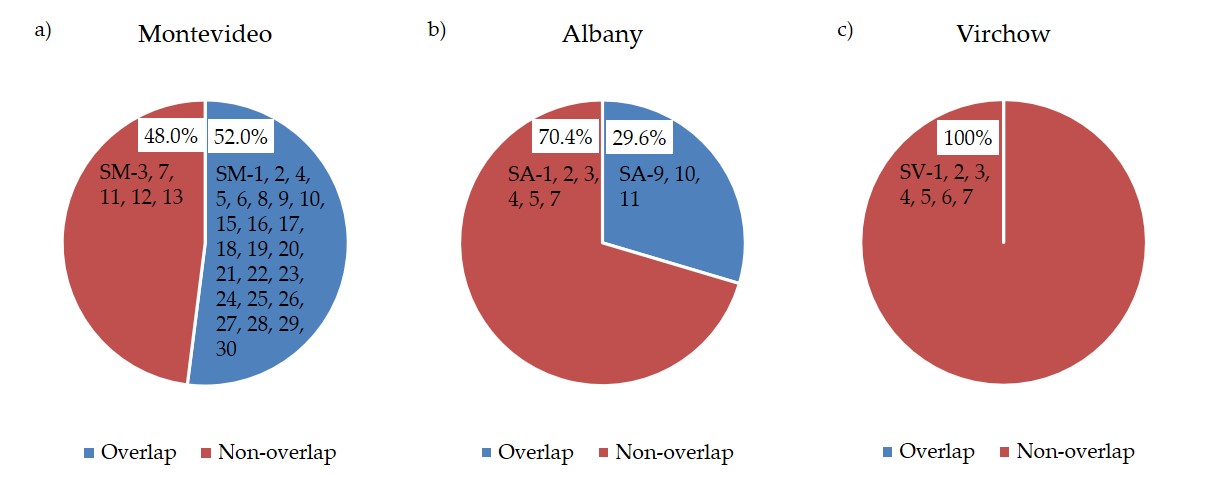


**Table S1.** Antimicrobials and the range of concentration tested.

| **Antimicrobial class** | **Antimicrobials** | **Abbreviation** | **Breakpoints (μg/mL)** | | | **Concentration range (μg/mL)** |
| --- | --- | --- | --- | --- | --- | --- |
|  |  |  | **S ^a^** | **I** | **R** |  |
| Quinolones | Nalidixic acid | NAL | 16 | - ^b^ | 32 | 2−128 |
| Fluoroquinolones | Ciprofloxacin | CIP | 0.06 | 0.12−0.5 | 1 | 0.12−16 |
|  | Enrofloxacin | ENR | 0.25 | 0.5−1 | 2 | 0.12−64 |
| Aminoglycosides | Neomycin | NEO | - | - | 16 | 2−32 |
|  | Gentamicin | GEN | 4 | 8 | 16 | 1−64 |
|  | Streptomycin | STR | 32 | - | 64 | 2−128 |
| Tetracyclines | Tetracycline | TET | 4 | 8 | 16 | 2−128 |
| β-lactams | Amoxicillin/clavulanic acid | AMC | 8/4 | 16/8 | 32/16 | 2/1−64/32 |
|  | Cephalexin | CEP | 16 | - | 32 | 2−64 |
|  | Cefoxitin | FOX | 8 | 16 | 32 | 1−32 |
|  | Ceftiofur | XNL | 2 | 4 | 8 | 0.5−8 |
|  | Ampicillin | AMP | 8 | 16 | 32 | 2−32 |
| Sulfonamides | Trimethoprim/sulfamethoxazole | SXT | 2/38 | - | 4/76 | 0.12/2.38−4/76 |
| Polymyxin | Colistin | COL | - | 2 | 4 | 2−32 |
| Phenicols | Florfenicol | FFN | - | - | 32 | 2−64 |
|  | Chloramphenicol | CHL | 8 | 16 | 32 | 2−64 |

^a^ S, sensitivity; I, intermediate resistance; R, resistance; ^b^, no standard breakpoint value in related reference

**Table S2.** Prevalence of *Salmonella* *enterica* in hatcheries and their upstream breeder farms ^*^.

| **Sample source** | **No. of samples** | **No. of isolates** | **Isolation rate (%)** |
| --- | --- | --- | --- |
| Hatchery | 220 | 36 | 16.4 ^a^ |
| Breeder farm | 200 | 6 | 3.0 ^b^ |
|  | 125 (S) | 1 | 0.8 ^b^ |
|  | 75 (L) | 5 | 6.7 ^b^ |
| Total | 420 | 42 | 10.0 |

^*^ S and L indicate cloaca swab and litter samples, respectively. Lowercase (a/b) were used to indicate significant difference in isolation rates among different sample sources and types; different letters indicate significant differences (*P* < 0.05)

**Table S3.** Prevalence of *Salmonella* *enterica* per hatcheries and breeder farms ^*^.

| **Source** | **No.** | **Farm** | **No. of samples** | **No. of isolates** | **Prevalence (%)** |
| --- | --- | --- | --- | --- | --- |
| Breeder farm | 1 | BY | 5 | 3 | 60.0 |
|  | 2 | ZE | 5 | 2 | 40.0 |
|  | 3 | NL | 5 | 1 | 20.0 |
| Hatchery | 1 | HNL | 5 | 4 | 80.0 |
|  | 2 | YY | 5 | 3 | 60.0 |
|  | 3 | HS | 5 | 3 | 60.0 |
|  | 4 | SJ | 5 | 3 | 60.0 |
|  | 5 | JZ | 5 | 2 | 40.0 |
|  | 6 | YWG | 5 | 2 | 40.0 |
|  | 7 | ZH | 5 | 2 | 40.0 |
|  | 8 | NS | 5 | 2 | 40.0 |
|  | 9 | YJ | 5 | 2 | 40.0 |
|  | 10 | SW | 5 | 2 | 40.0 |
|  | 11 | NSZY | 5 | 2 | 40.0 |
|  | 12 | XH | 5 | 2 | 40.0 |
|  | 13 | YG | 5 | 2 | 40.0 |
|  | 14 | JHG | 5 | 1 | 20.0 |
|  | 15 | YW | 5 | 1 | 20.0 |
|  | 16 | HSH | 5 | 1 | 20.0 |
|  | 17 | BY | 5 | 1 | 20.0 |
|  | 18 | XN | 5 | 1 | 20.0 |

^*^ Three of 25 (12.0%) breeder farms were positive with *Salmonella enterica*; eighteen of 44 hatcheries (40.9%) were positive with *Salmonella*.

**Table S4.** The prevalence of *Salmonella* isolates from downstream of the hatchery with overlap the PFGE types with the hatchery stage ^*^.

| **Serovar (*n*)** | **Overlapped PFGE types** | |  | **PFGE types with overlap or not** | | | |
| --- | --- | --- | --- | --- | --- | --- | --- |
|  | ***n* (%)** | ***p* value** |  | **Overlap (*n*)** | **Leading type** | ***n* (%)** | ***p* value** |
| Montevideo (*n* = 100) | 52 (52.0%) | 0.003 |  | Yes (*n*= 52) | SM-7 | 25 (48.1%) | < 0.001 |
|  |  |  |  | No (*n*= 48) | SM-6 | 4 (8.3%) |  |
| Albany  (*n* = 71) | 21 (29.6%) |  |  | Yes (*n*= 21) | SA-11 | 14 (66.7%) | 0.018 |
|  |  |  |  | No (*n*= 50) | SA-4 | 24 (48.0%) |  |

^*^ The chi-square test was used to test for significant differences of the prevalence of *Salmonella* isolates overlapped PFGE patterns between different serotypes; statistical difference between the most prevalent overlapped and non-overlapped PFGE types in each serotype; *p* values less than 0.05 were considered statistically significant. ’Overlap’ indicates same PFGE types shared between hatchery and its downstream stages; *n*, no. of isolates; %, prevalence
