# Supplementary figures and images for: The Occurrence of Antimicrobial-Resistant Salmonella enterica in Hatcheries and Dissemination in an Integrated Broiler Chicken Operation in Korea
Source: Animals (Basel). 2021 Jan 11;11(1):154. doi: 10.3390/ani11010154 (PMC7827806; doi:10.3390/ani11010154)

(a)

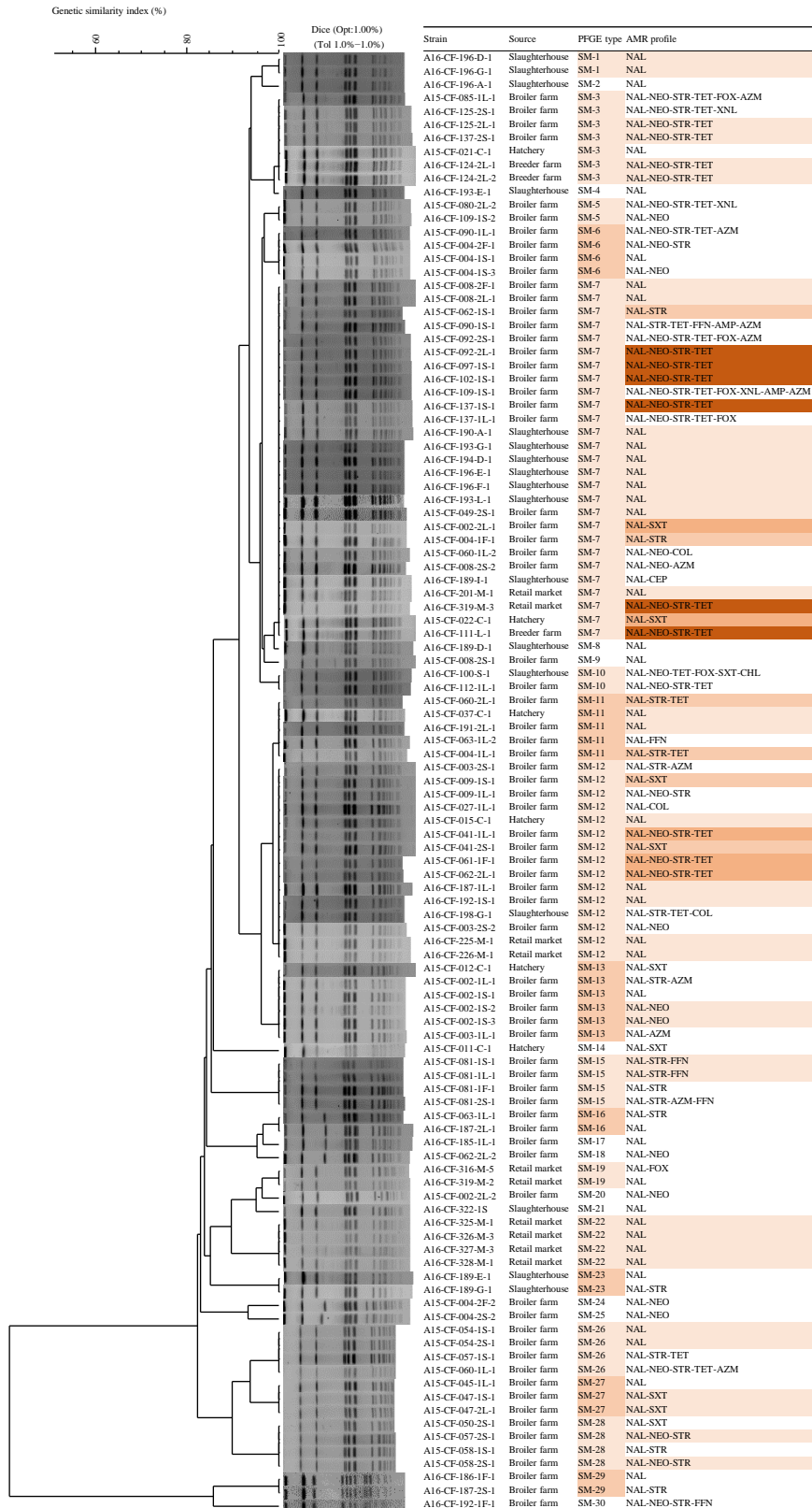

(b)

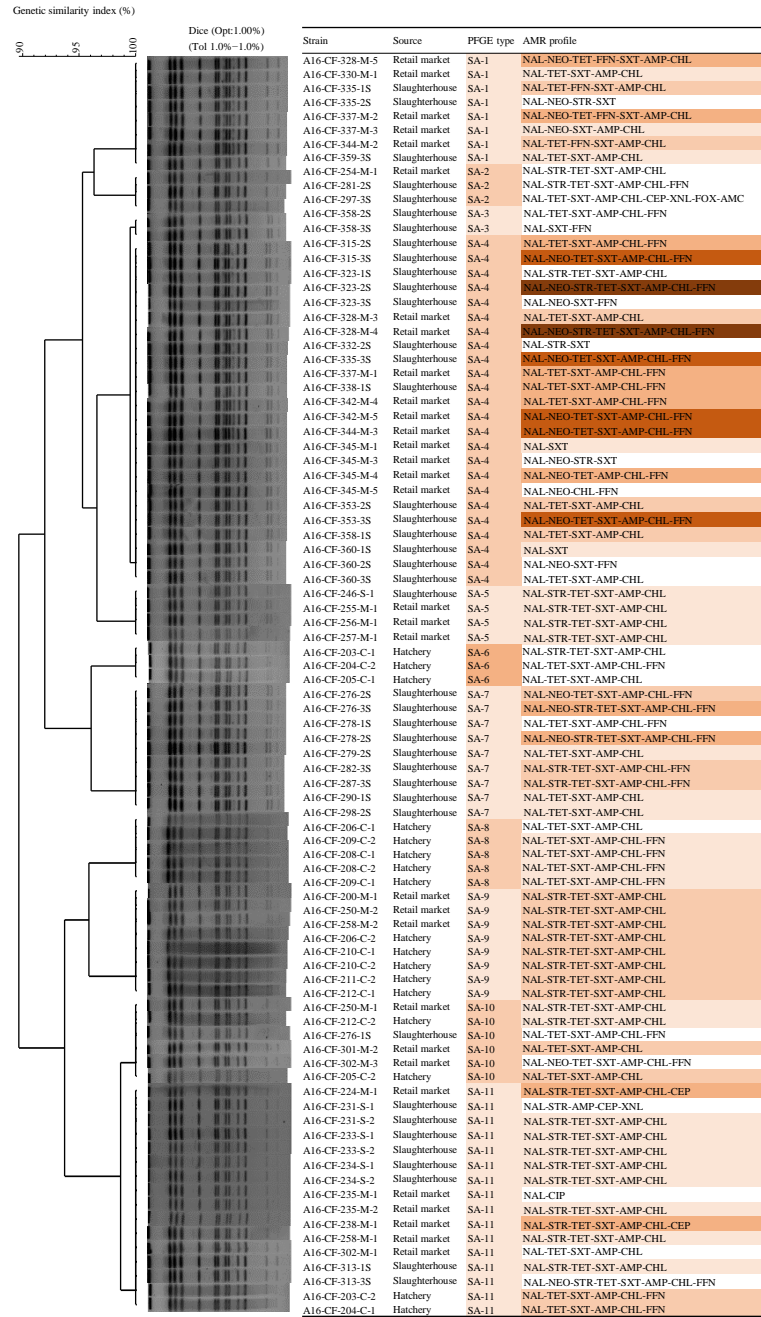

(c)

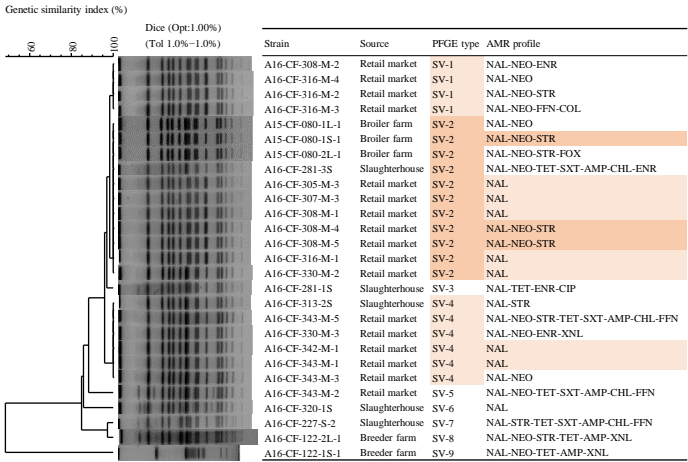

Supplement: Supplementary file 1 [file animals-11-00154-s001.zip › supple/Figure S1.pdf]
